# PEITH(Θ): perfecting experiments with information theory in Python with GPU support

**DOI:** 10.1093/bioinformatics/btx776

**Published:** 2017-12-07

**Authors:** Leander Dony, Jonas Mackerodt, Scott Ward, Sarah Filippi, Michael P H Stumpf, Juliane Liepe

**Affiliations:** 1 Department of Life Sciences, Imperial College London, London, UK; 2 Faculty of Medicine, School of Public Health, Imperial College London, London, UK; 3 Department of Mathematics, Imperial College London, London, UK; 4 Max-Planck-Institute for Biophysical Chemistry, Göttingen, Germany

## Abstract

**Motivation:**

Different experiments provide differing levels of information about a biological system. This makes it difficult, *a priori*, to select one of them beyond mere speculation and/or belief, especially when resources are limited. With the increasing diversity of experimental approaches and general advances in quantitative systems biology, methods that inform us about the information content that a given experiment carries about the question we want to answer, become crucial.

**Results:**

PEITH(Θ) is a general purpose, Python framework for experimental design in systems biology. PEITH(Θ) uses Bayesian inference and information theory in order to derive which experiments are most informative in order to estimate all model parameters and/or perform model predictions.

**Availability and implementation:**

https://github.com/MichaelPHStumpf/Peitho

## 1 Introduction

Quantitative descriptions of biological systems are being used ever more frequently, helping us to understand them in detail whilst also providing a framework to make predictions on their future behaviour (Liepe *et al.*, 2015). Popular modelling approaches include ordinary (ODEs) and stochastic (SDEs) differential equations, where parameters are commonly inferred from experimental data (Liepe *et al.*, 2010, 2014). Conducting more and better experiments can improve the inference of parameters and result in better, more accurate models. The design of the experiments is, however, often neglected, despite the fact that this can dramatically alter the insights that can be gained about a system’s behaviour: data collected from different experiments do not carry equal information, and some experiments may yield negligible new information. The question of how we can pick optimal experiments to best infer parameters or make predictions about a system’s behaviour is thus of pivotal importance.

Experimental design within an information theoretic framework has been explored previously (Barnes *et al.*, 2011; Liepe *et al.*, 2013; Vanlier *et al.*, 2012) but a user-friendly package to conduct the design of experiments has yet been lacking. Here we implement algorithms for experimental design in order to obtain reliable parameter estimates as well as model predictions. The algorithms combine concepts from Bayesian inference and information theory in order to identify experiments that maximize the information content of the resulting data as described in (Liepe *et al.*, 2013). Below we will introduce the main concepts underlying the algorithms and we provide a package overview. A detailed explanation of all algorithms and a comprehensive step-by-step guide with examples can be found in the package manual available via the github repository.

## 2 Materials and methods

The package PEITH(Θ) is implemented as a Python package and is available as *peitho* through the PyPI. PEITH(Θ) makes use of the python package CUDA-sim (Zhou *et al.*, 2011) for parallel fast model simulations.

### 2.1 PEITH(Θ) for inference of model parameters and for prediction

In a Bayesian framework model parameters are treated as random variables for which we can define a prior probability distribution and the aim is often to infer the posterior probability distribution after having observed some data. The reduction in uncertainty of the model parameters arising from gathering data during an experiment can be measured by the mutual information between model parameters and the possible experimental outcomes, which is equal to the difference in entropy between the prior and the posterior probability distributions. In order to determine the optimal experiment to infer the model parameters one can maximize this mutual information. To compute the latter we derive the integrals in (Liepe *et al.*, 2013). This gives, for each experiment a measure of the average reduction in uncertainty for the model parameters, thus providing an approach to quantitatively capture those experiments that provide *substantial* and *relevant* information (Liepe *et al.*, 2013).

We extend this concept to reduce the uncertainty in the estimation of a subset of parameters. This is particular relevant, because the experiment that is optimal to estimate all systems parameters is often not the best to estimate a specific parameter with high certainty. Related to this, one often wants to predict an experiment that cannot feasibly be carried out. In this case the experiment that is most suited to infer all model parameters is not always the most informative to obtain predictions with high certainty since some specific parameters might drive the model behaviour.

PEITH(Θ) implements algorithms for all three described scenarios: reducing the uncertainty of the model parameters, a subset of the parameters, or the prediction of the outcome on an intervention. More often than not the resulting integrals are not analytically tractable and require numerical estimation, which we do here using Monte Carlo estimates (Liepe *et al.*, 2013). This in turn is computationally extremely expensive and for most systems of biological relevance prohibits the calculation on CPUs. We therefore implemented and optimized both numerical model simulations [using CUDA-sim (Zhou *et al.*, 2011)] and all Monte Carlo estimations on GPUs.

### 2.2 Package overview and options

**Fig. 1.**
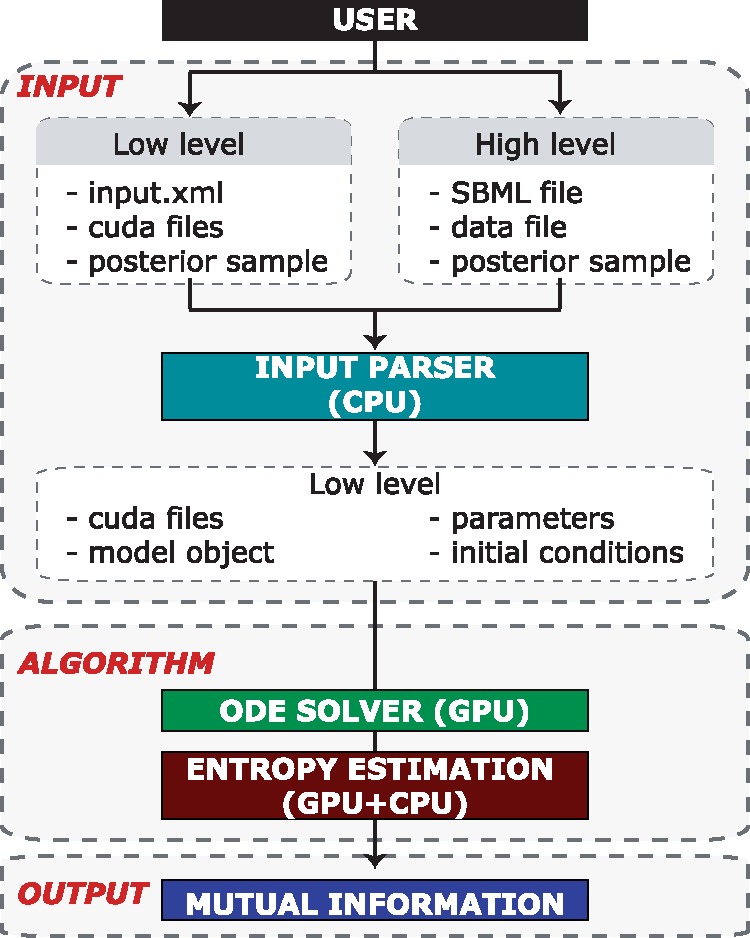
Schematic representation of PEITH(Θ) overall package structure. PEITH(Θ) is separated into 3 parts: *Input*, *Algorithm* and *Output*

PEITH(Θ) can be separated into three major components: *Inputs*, *Algorithms* and *Output* (Fig. 1); however, the user will only interact with the *Input* component. We have developed two pipelines, and their usage depends on the user’s computational background. The high-level automated input pipeline, which caters to those with less computational experience, takes an SBML model file with an additional data file highlighting how experiments can be conducted. However, this pipeline is limited to those biological systems that can be defined through the SBML model file format. The alternative approach is the low-level, more involved input pipeline, which takes as input an.xml file and a manually written CUDA code file, which follows the standard *CUDA-sim* format (Zhou *et al.*, 2011). This gives computationally experienced users the ability to define more complex biological systems and supports events and rules during model simulation. The *Output* component presents the estimated mutual information and additional algorithm to the user, which are all stored in a standard *csv* format.

## 3 Summary

By using information theory PEITH(Θ) provides a systematic approach to recommending potential experiments beyond belief and speculation. It allows users to define a variety of potential experiments from varying the time points to different initial conditions and with both high level and low level pipelines it caters to a wide demographic of computational experiences. Together with the Python packages *ABC-SysBio* and *CUDA-sim* we now provide all tools for experimental design, model development and parameter estimation in a Bayesian framework.
